# Remote Sensing Target Tracking Method Based on Super-Resolution Reconstruction and Hybrid Networks

**DOI:** 10.3390/jimaging11020029

**Published:** 2025-01-21

**Authors:** Hongqing Wan, Sha Xu, Yali Yang, Yongfang Li

**Affiliations:** School of Mechanical and Automotive Engineering, Shanghai University of Engineering Science, 333 Longteng Road, Songjiang District, Shanghai 201620, China; m310122474@sues.edu.cn (H.W.); yyl@sues.edu.cn (Y.Y.); liyongfang@sues.edu.cn (Y.L.)

**Keywords:** remote sensing image, super-resolution reconstruction, target tracking, CNN, GRU

## Abstract

Remote sensing images have the characteristics of high complexity, being easily distorted, and having large-scale variations. Moreover, the motion of remote sensing targets usually has nonlinear features, and existing target tracking methods based on remote sensing data cannot accurately track remote sensing targets. And obtaining high-resolution images by optimizing algorithms will save a lot of costs. Aiming at the problem of large tracking errors in remote sensing target tracking by current tracking algorithms, this paper proposes a target tracking method combined with a super-resolution hybrid network. Firstly, this method utilizes the super-resolution reconstruction network to improve the resolution of remote sensing images. Then, the hybrid neural network is used to estimate the target motion after target detection. Finally, identity matching is completed through the Hungarian algorithm. The experimental results show that the tracking accuracy of this method is 67.8%, and the recognition identification F-measure (IDF1) value is 0.636. Its performance indicators are better than those of traditional target tracking algorithms, and it can meet the requirements for accurate tracking of remote sensing targets.

## 1. Introduction

Super-resolution reconstruction and target tracking represent two pivotal research directions in the field of computer vision. The combination of the two technologies has significant value in the fields of video surveillance, autonomous driving, and remote sensing. Target tracking aims to model the target tracking process in video sequences. Precise target tracking can be applied in scenarios such as intelligent video analysis and personnel tracking [1]. With the wide application of deep learning technologies, the reconstruction performance from low-resolution images to high-resolution images has been significantly improved [2,3]. This is of great significance for the target tracking task, as it can help algorithms identify abundant target features more precisely [4,5] and further improve the accuracy and robustness of the algorithms.

Super-resolution reconstruction algorithms can be divided into two categories: traditional multi-image super-resolution algorithms and single-image super-resolution algorithms [6,7]. Many scholars at home and abroad have conducted in-depth explorations in the field of super-resolution reconstruction technology. On the one hand, in the research on super-resolution reconstruction methods based on deep learning, they have actively explored neural network architectures applicable to remote sensing images [8,9]. For example, some research teams have improved and optimized traditional convolutional neural networks (CNNs) and generative adversarial networks (GANs) in light of the characteristics of remote sensing images to enhance the effect of super-resolution reconstruction [10]. On the other hand, there have also been quite a number of research achievements in the super-resolution reconstruction of multi-source remote sensing images. They are committed to solving the problems of registration, fusion, etc., in multi-source data fusion so as to make full use of the complementary information of multi-source remote sensing data and improve the quality and accuracy of the reconstructed images [11]. The tracking effect of super-resolution reconstruction technology under complex environmental conditions (such as clouds, fog, atmospheric interference, etc.) and for complex targets (such as small targets, camouflaged targets, etc.) is constantly being improved by optimizing the algorithm to enhance its adaptability to various complex situations [12]. On the premise of ensuring the accuracy of super-resolution reconstruction and target tracking, research is being conducted on how to improve the running speed and efficiency of algorithms to meet the requirements for real-time performance in practical applications [13]. With the continuous development of deep learning in the field of computer science, it will also bring more inspiration to the field of super-resolution.

Traditional super-resolution reconstruction algorithms have certain limitations and fail to adequately address the challenges posed by environmental complexity and noise in remote sensing images, resulting in suboptimal reconstruction outcomes. Therefore, this paper proposes a super-resolution reconstruction algorithm specifically designed for remote sensing images to fully capture the detailed features of remote sensing images and improve the model’s accuracy. In addition, general target tracking algorithms often over-emphasize the appearance similarity between targets across frames while neglecting the nonlinear characteristics of target motion. To address this, this paper designs a hybrid neural network, CNN-GRU, that extracts both local features and global information, allowing the target tracking algorithm to more effectively consider the nonlinear characteristics of target motion, thus improving tracking accuracy. The target tracking algorithm proposed in this paper, specifically for remote sensing images, is suitable for detecting small and camouflaged targets, offering advantages such as high accuracy and strong applicability. Compared to traditional target tracking and super-resolution reconstruction algorithms, the methods proposed in this paper show significant improvements and offer substantial advantages.

## 2. Overall Framework of Remote Sensing Target Tracking System

**Figure 1:**
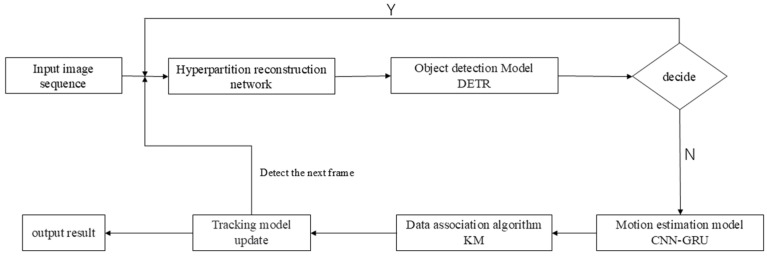
Overall system framework.

The overall framework of the system is shown in Figure 1. First, the input remote sensing image sequences undergo super-resolution reconstruction using the improved network to enhance image quality. Next, the reconstructed images are fed into the target detection model, detection transformer (DETR) [14], based on the transformer framework, which outputs the detected remote sensing targets. Then, the detection boxes are passed into the motion estimation model for motion modeling and state estimation, followed by the calculation of the cost matrix. Finally, the Hungarian algorithm [15] is applied to perform inter-frame target data association and identity transfer, updating the tracking model and outputting the tracking result.

## 3. Main Algorithms

### 3.1. Super-Resolution Reconstruction Algorithm

General super-resolution reconstruction models are mainly used for processing natural landscapes and cannot fully take into account characteristics such as the complexity of scenes and noise in remote sensing images, which leads to poor reconstruction effects. Therefore, based on EDSR [16], this paper proposes a super-resolution reconstruction model specifically designed for remote sensing images. This network has redesigned the residual blocks, upsampling blocks, and loss functions, improving the model’s feature extraction ability and further enhancing the details and texture of the reconstructed images.

#### 3.1.1. Residual Block

Deepening the residual blocks can effectively enhance the network’s ability to extract image features. For this reason, this paper proposes a residual block based on the convolutional layer, the GELU activation function [17], and the BN layer. The convolutional layer adopts small-scale convolutional kernels to better handle the local features of remote sensing images and increase the receptive field. Meanwhile, the GELU activation function is added after each convolutional layer. The GELU is a continuous and smooth function, and its derivative exists throughout the entire real number domain, which can avoid the vanishing gradient phenomenon during the training process and accelerate convergence. The formula is:

(1) $$GELU\left(x\right)=0.5x\left\{1+\tanh\left[\sqrt{2/\pi }\left(x+0.044715x^{3}\right)\right]\right\}$$

where x is the input feature.

**Figure 2:**
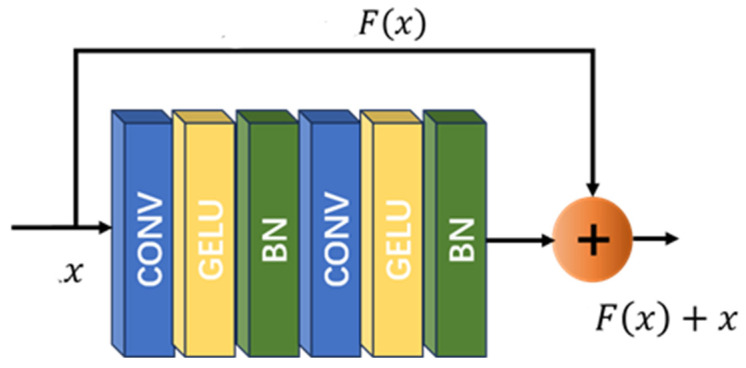
Residual module network structure.

The network structure of the residual module is shown in Figure 2, where the BN layer is used to accelerate model convergence.

#### 3.1.2. Upsampling Block

The upsampling block proposed in this paper is alternately composed of a deconvolution layer [18] and a sub-pixel convolution layer. The deconvolution layer achieves upsampling by mapping each pixel of the input tensor to multiple pixels of the output tensor, which can reduce phenomena such as jagged distortion in remote sensing images. The sub-pixel convolution layer improves the quality of the reconstructed image by reducing the number of channels and increasing the resolution.

Let the input size be $\left(B,C,H_{i},W_{i}\right)$ (number of batches, number of channels, height, width, respectively) and the deconvolution calculation formula be

(2) $$M_{o}=\frac{M_{i}+2p-k}{s}+1$$

where *M* is the height or width, *p* is the fill number, *k* is the convolution kernel size, *s* is the convolution kernel size, and *i* and *o* represent the input and output. After the deconvolution operation, the size of the image is transformed into $\left(B,C*r^{2},H_{o},W_{o}\right)$, and *r* is the magnification.

Sub-pixel convolution rearranges and reorganizes the image after deconvolution operation according to certain rules. In the first step, the input tensor is divided according to the number of channels to obtain $r^{2}$ sub-tensors, each of which has dimensions of $\left(B,C,H_{o},W_{o}\right)$. In the second step, each subtensor is rearranged and reorganized. First, a new tensor of size r∗r is constructed, and then the height and width of each subtensor are scaled to 1/*r*. Finally, the scaled subtensor is filled into the construction tensor row by row and column by pixel. In the third step, $r^{2}$ new tensors are concatenated according to channel dimension to obtain an output tensor of $\left(B,C,H_{o}*r,W_{o}*r\right)$.

#### 3.1.3. Loss Function

The loss function is composed of mean squared error (MSE) [19] and structural similarity index measure (SSIM) [20] loss function, calculated by the formula:

(3) $$loss=loss_{MSE}+loss_{SSIM}$$

MSE is an indicator for measuring image quality. By calculating the differences between two images at corresponding pixel points, it can effectively capture information such as noise and distortion in remote sensing images. The calculation formula is as follows:

(4) $$loss_{MSE}=\frac{1}{mn}\overset{m-1}{\underset{i=0}{\sum }}\overset{n-1}{\underset{j=0}{\sum }}{\left[X\left(i,j\right)-Y\left(i,j\right)\right]}^{2}$$

In the formula, *m* and *n* are the number of pixels in the high and wide directions of the input image, $\left(i,j\right)$ are the pixel coordinates, and *X* and *Y* represent the super-resolution reconstructed image and the corresponding label image, respectively.

The SSIM loss function can effectively handle high-level features such as the structure and texture of remote sensing images. It reflects the image reconstruction quality by examining luminance, contrast, and structure. The calculation formula is as follows:

(5) $$\left\{\begin{array}{c} L\left(X,Y\right)=\frac{2\mu_{x}\mu_{y}+C_{1}}{\mu_{x}^{2}+\mu_{y}^{2}+C_{1}}\\ C\left(X,Y\right)=\frac{2\sigma_{x}\sigma_{y}+C_{2}}{\sigma_{x}^{2}+\sigma_{y}^{2}+C_{2}}\\ S\left(X,Y\right)=\frac{\sigma_{xy}+C_{3}}{\sigma_{x}\sigma_{y}+C_{3}} \end{array}\right.$$

where *L*, *C*, and *S* are the values of brightness, contrast, and structure, respectively. μ is the mean value of the pixel, σ is the variance of the pixel value, and $C_{1},C_{2},C_{3}$ are constants. In summary, the calculation formula of SSIM loss function is as follows:

(6) $$loss_{SSIM}=\frac{\left(2\mu_{x}\mu_{y}+C_{1}\right)\left(2\sigma_{xy}+C_{2}\right)}{\left(\mu_{x}^{2}+\mu_{y}^{2}+C_{1}\right)\left(\sigma_{x}^{2}+\sigma_{y}^{2}+C_{2}\right)}$$

### 3.2. Motion Estimation

#### 3.2.1. Estimation Model

After the remote sensing images have been detected, it is necessary to model the detected targets to reflect their inter-frame motion states so that the identities of the targets can be propagated to the next frame. Let the state of each remote sensing target be:

(7) $$K={\left[u,v,\gamma ,h,\overset{\cdot }{u},\overset{\cdot }{v},\overset{\cdot }{\gamma ,}\overset{\cdot }{v}\right]}^{T}$$

In the formula, u and v represent the horizontal and vertical pixel positions of the target center, γ and h represent the aspect ratio and height of the detection box, and the remaining variables are the first derivative of the corresponding variables

#### 3.2.2. Hybrid Neural Network

**Figure 3:**
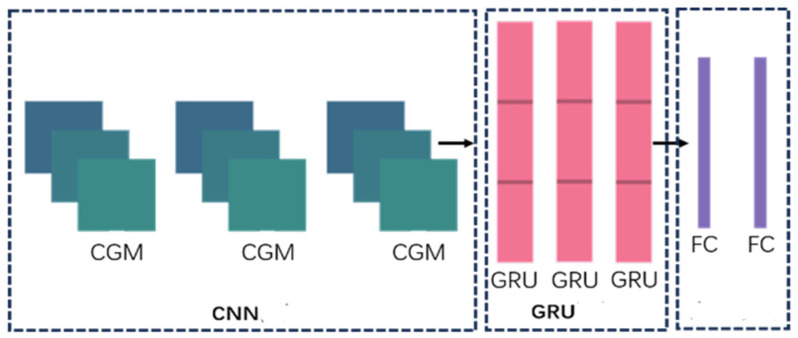
Hybrid neural network structure.

Remote sensing targets have characteristics such as variable positions and large variations in size. This makes it impossible for traditional tracking algorithms to represent the nonlinearity of the motion of remote sensing targets when the speed is directly set as a constant, resulting in inaccurate target state estimation and exacerbating the accumulation of errors. Therefore, this paper proposes a hybrid neural network called CNN-GRU. It regards the motion of remote sensing targets between frames as a time series prediction problem. The model is composed of a convolutional neural network (CNN) [21] and a gated recurrent unit (GRU) [22], which can extract local information and the context of sequential data, thus effectively capturing the nonlinear characteristics of the motion between frames. Its structure is shown in Figure 3.

Let the input of CNN-GRU be matrix *P*; the vertical direction of the matrix represents the state of each target identity ${\left[u,v,\gamma ,h,\overset{\cdot }{u},\overset{\cdot }{v},\overset{\cdot }{\gamma ,}\overset{\cdot }{v}\right]}^{T}$, the horizontal direction represents the state quantity corresponding to the first ten frames of images, and matrix *P* is

(8) $$P=\left[\begin{array}{ccc} \begin{array}{cc} u_{t-10} & u_{t-9}\\ v_{t-10} & v_{t-9} \end{array} & \cdots & \begin{array}{c} u_{t-1}\\ v_{t-1} \end{array}\\ \vdots & \ddots & \vdots \\ \begin{array}{cc} {\dot{h}}_{t-10} & {\dot{h}}_{t-9} \end{array} & \cdots & {\dot{h}}_{t-1} \end{array}\right]$$

where t represents the current frame. At this time, the hybrid neural network outputs the predicted value Q of the target identity state in the current frame, i.e.,:

(9) $$Q={\left[u_{t},v_{t},\gamma_{t},h_{t},\overset{\cdot }{u_{t}},\overset{\cdot }{v_{t}},\overset{\cdot }{\gamma_{t}},\overset{\cdot }{h_{t}}\right]}^{T}$$

The CNN is used to extract the local features of remote sensing target trajectory data, making up for the deficiency that the recurrent neural network can only focus on the contextual information globally. The CNN has three CGM modules, and each module is composed of a 1D convolutional layer, a GELU activation function, and a 1D max pooling layer. Among them, the 1D convolutional layer is specifically designed to handle time series data, and its convolutional kernels slide only in the horizontal direction.

**Figure 4:**
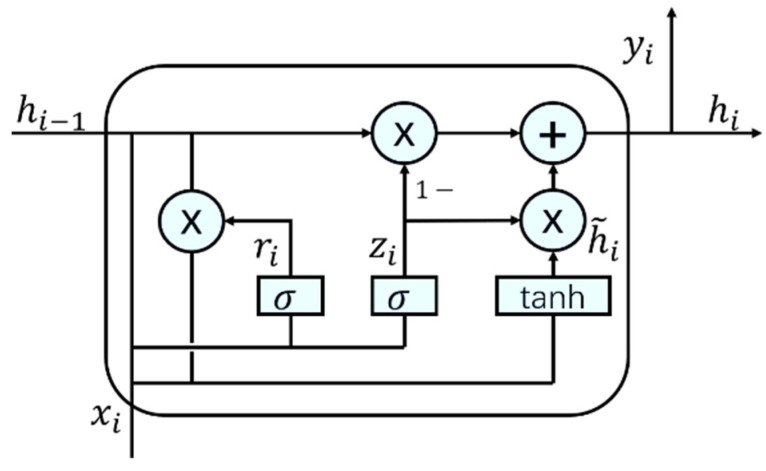
Gated cycle unit, GRU.

GRU is a special recurrent neural network structure. It can solve problems such as the vanishing gradient and exploding gradient to some extent during time series training. Meanwhile, it also has a certain degree of memory and can capture the long-term dependencies in sequential data. This paper adopts a three-layer GRU network; this is used to capture the temporal information in the high-level features after convolutional calculations. The GRU contains a reset gate and an update gate, and its structure is shown in Figure 4. In the figure, $h_{i-1}$ and $h_{i}$ are the passed hidden layer state, ${\tilde{h}}_{i}$ is the candidate state after reset, $r_{i}$ and $z_{i}$ are the reset gate and update gate, respectively. And $x_{i}$ and $y_{i}$ are the input and output of node i, respectively.

The status update formula of the gated cycle unit is:

(10) $$r_{i}=\sigma \left(W_{r}x_{i}+U_{r}h_{i-1}+b_{r}\right)$$

(11) $$z_{i}=\sigma \left(W_{z}x_{i}+U_{z}h_{i-1}+b_{z}\right)$$

The status update formula for the memory unit is:

(12) $${\tilde{h}}_{i}=\tan h\left(W_{h}x_{i}+U_{h}\left(r_{i}⊙h_{i-1}\right)+b_{h}\right)$$

(13) $$h_{i}=z_{i}⊙h_{i-1}+(1-z_{i})⊙{\tilde{h}}_{i}$$

This node outputs the formula as:

(14) $$y_{i}=\sigma \left(W_{y}h_{i}\right)$$

In the formula, $W_{r},W_{z},W_{h},W_{y}$ are input weight vectors of reset gate, update gate, candidate unit, and output gate, respectively. $U_{r},U_{z},U_{h}$ are the cyclic weight vectors of the corresponding units, respectively. $b_{r},b_{z},b_{h},b_{y}$ are input weight vectors of reset gate, update gate, candidate unit, and output gate, respectively. σ is the Sigmoid activation function [23], whose expression is:

(15) $$\sigma \left(x\right)=\frac{1}{1+e^{-x}}$$

### 3.3. Data Association

After motion estimation, it is necessary to conduct data association between the two frames before and after the motion estimation and to complete identity transfer and a status update. After the remote sensing image is detected, detection boxes are generated, and each detection box has four variables: *u*, *v*, *γ*, and h. If the remote sensing image of frame t-1 has target frames, each detection frame has target frames to match, and the number for matching is m∗n. This article will regard the time interval between two frames as constant Δt, the match after each measured value *D* for ${\left[u_{t},v_{t},\gamma_{t},h_{t},\overset{\cdot }{u_{t}},\overset{\cdot }{v_{t}},\overset{\cdot }{\gamma_{t}},\overset{\cdot }{h_{t}}\right]}^{T}$.

In this paper, the box represented by the four variables of the predicted value *Q* of frame t and the measured value u,v,γ,h is calculated in pairs to measure the position similarity between the predicted value and the measured value. The formula is as follows:

(16) $$r_{IoU}=\frac{D_{l}\cap Q_{l}}{D_{l}\cup Q_{l}}$$

where l represents the quantity of position.

The four variables $\overset{\cdot }{u},\overset{\cdot }{v},\overset{\cdot }{\gamma ,}\overset{\cdot }{v}$ in the predicted value *Q* of frame t and the measured value *D* are calculated to reflect the velocity similarity between the measured value and the measured value by solving the Mahalanobis distance. The larger the Mahalanobis distance, the more dissimilar the two are. The formula is as follows:

(17) $$r_{M}={\left(D-Q\right)}^{T}S^{-1}\left(D-Q\right)$$

where S is the covariance of the predicted value *Q* and the measured value *D*.

Use the weighted values of the two as the total similarity, and calculate the formula as follows:

(18) $$r=\lambda r_{IoU}+\left(1-\lambda \right)r_{M}$$

where λ represents the weight; the larger the value of r, the more similar the predicted value *Q* is to the measured value *D*, and the range of R is [0, 1].

At this time, the calculated cost matrix is $\left(\begin{array}{ccc} \begin{array}{cc} r_{11} & r_{12}\\ r_{21} & r_{22} \end{array} & \cdots & \begin{array}{c} r_{1n}\\ r_{2n} \end{array}\\ \vdots & \ddots & \vdots \\ \begin{array}{cc} r_{m1} & r_{m2} \end{array} & \cdots & r_{mn} \end{array}\right)$; this should be input into the Hungarian algorithm in order to complete the target identity matching and status update.

## 4. Experiments and Analysis

### 4.1. Selection and Preprocessing of the Dataset

This paper adopts the DIV2K dataset [24] as the dataset for the super-resolution reconstruction model. This dataset contains 800 high-resolution images and their corresponding low-resolution images for training and validation and another 100 images for testing. Among them, the size of the low-resolution images is one-fourth that of the high-resolution images, respectively. Since remote sensing images are usually affected by various noises, this paper adds noise to the DIV2K dataset to better simulate the actual situation. The specific operations are as follows: by setting a signal-to-noise ratio of 0.05 and generating random numbers for pixel-by-pixel noise addition operations, salt-and-pepper noise is added to the training set; with a mean of 0 and a variance of 0.03, Gaussian perturbations are performed on the pixel values of the pictures in the training set. After enhancement, the training set and the validation set are three times the original size. Then, the training set, the validation set, and the test set are divided, with 2100, 300, and 100 images, respectively.

The VISO dataset is adopted as the dataset for the target detection and tracking models. This dataset contains four types of targets, namely ships, cars, trains, and airplanes. For the training set of target detection, random horizontal and vertical flips are performed with the proportion controlled at 3:7 to enhance the model’s ability to represent spatial positions. The RGB channels of the images are converted to HSV, and the perturbation coefficients for hue, saturation, and brightness are set to 0.02, 1.15, and 0.6, respectively. The tracking dataset is processed using the sliding window method with a size of 10 and a step length of 1. Therefore, the input feature dimension of the model is 8, the sequence length is 10, the output feature dimension is 8, and the sequence length is 1.

### 4.2. Experimental Platform and Parameter Settings

This paper adopts a cloud server system with the following configuration: Ubuntu 18.04 64-bit system; the CUDA version is 11.0.2; the GPU is NVIDIA T4; and the AIACC training acceleration and AIACC inference acceleration architectures are employed. During model training, the batch size is set to 32, the optimizer is Adam, and the learning rate is 0.0001. The weight *λ* for data association is solved as 0.674.

### 4.3. Results and Analysis of Super-Resolution Reconstruction

This paper employs two of the most commonly used metrics in the field of super-resolution, namely peak signal-to-noise ratio (PSNR) and structural similarity index measure (SSIM) [25], to evaluate the reconstruction effect of remote sensing images. PSNR is used to measure the differences between two images and assess the quality of super-resolution reconstructed images, as well as the performance of algorithms.

(19) $$PSNR=10\log_{10}(\frac{MaxValue^{2}}{MSE})$$

Among them, MSE represents the mean squared error of two images on a pixel-by-pixel basis, and MaxValue is the maximum value that an image pixel can be assigned.

The SSIM is an indicator used to measure the similarity between two images. It is based on three relatively independent subjective metrics, namely luminance, contrast, and structure. Compared with the traditional MSE, it is more in line with the perception of images by the human visual system.

(20) $$SSIM(x,y)=\frac{\left(2\mu_{x}\mu_{y}+c_{1})(2\sigma_{xy}+c_{2}\right)}{\left(\mu_{x}^{2}+\mu_{y}^{2}+c_{1})(\sigma_{x}^{2}+\sigma_{y}^{2}+c_{2}\right)}$$

Among them, $\mu_{x}$ is the mean value of *x*, $\sigma_{x}$ is the variance of x, x and y are N × N images of the same size, and $\sigma_{xy}$ is the covariance of x and y. $c_{1}={\left(k_{1}L\right)}^{2}$, $c_{2}={\left(k_{2}L\right)}^{2}$, and $c_{3}=c_{2}/2$ are constants, L is the range of pixel values, $k_{1}$ and $k_{2}$ are constants much smaller than 1. By default, $k_{1}$ = 0.01, and $k_{2}$ = 0.03.

The ablation experiment is an experimental method widely used in the fields of machine learning, deep learning, etc. Its main purpose is to analyze the contribution degree of each component (such as network layers, features, modules, etc.) in a complex model to the overall performance of the model. In this way, it can quantitatively characterize the improvement before and after network improvement and the contribution of each component to the whole. The results of the ablation experiment are shown in Table 1. Improvement 1 redesigned the residual module, with the peak signal-to-noise ratio (PSNR) and structural similarity index measure (SSIM) increased by 0.38 and 0.086, respectively, indicating that appropriately deepening the network can fully extract image features. Improvement 2 set the upsampling block to alternate calculations between the deconvolution layer and the sub-pixel convolution layer, which has higher accuracy in expanding the model and filling pixels than the upsampling block that only adopts the deconvolution layer. Improvement 3 added the SSIM loss function, which reflects the performance of the model from three aspects: luminance, contrast, and structure. Therefore, compared with the traditional EDSR model, the super-resolution reconstruction network proposed in this paper has been greatly improved in processing remote sensing images; among them, the PSNR and SSIM indicators have been increased by 1.17 dB and 0.12, respectively.

### 4.4. Analysis of Target Tracking Results

**Figure 5:**
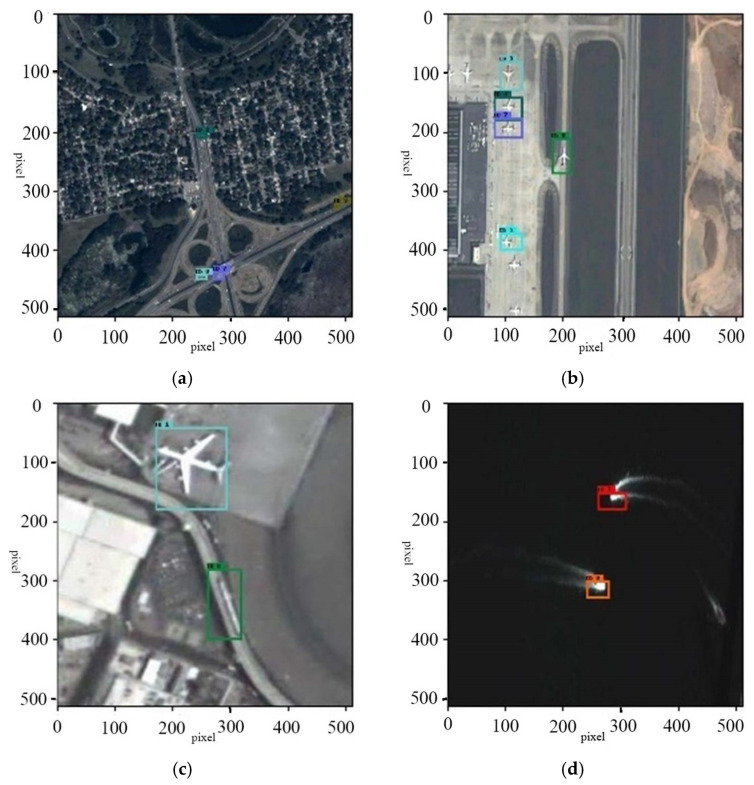
Target tracking effect without overshoot reconstruction.

**Figure 6:**
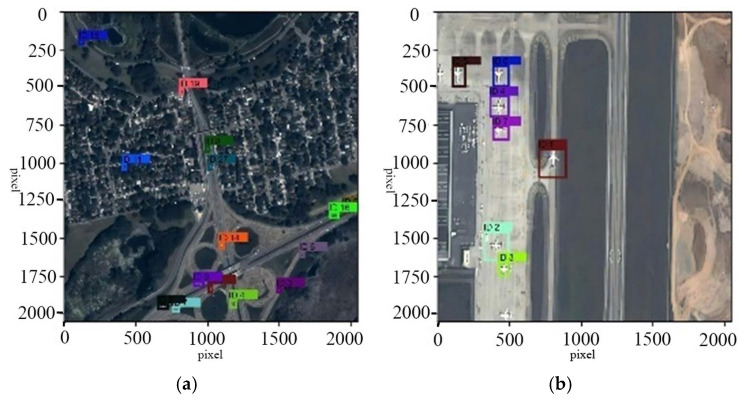

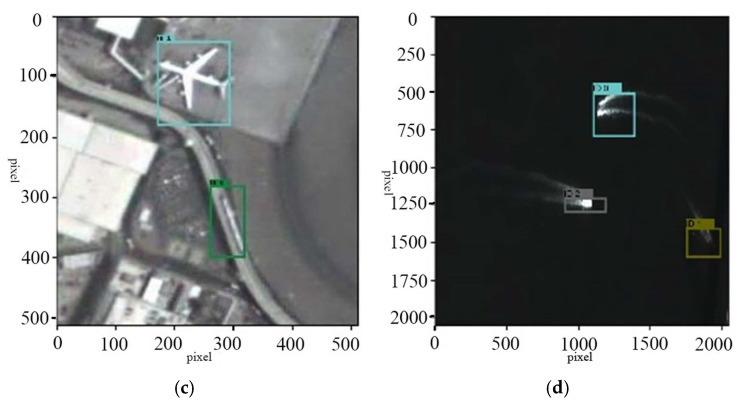
Target tracking effect after overshoot reconstruction.

The image sequences in the VISO dataset are input into the model for testing. Parts of the testing results are shown in the Figure 5 and Figure 6. The upper and lower parts, respectively, display the effects of remote sensing target tracking before and after super-resolution reconstruction. The tracked targets are marked by rectangular boxes, and their current identities are shown.

To describe the effect of the tracking model more precisely, this paper adopts two metrics, namely multiple object tracking accuracy (MOTA) and IDF1, to analyze the tracking accuracy of the model. Among them, MOTA is a widely used evaluation metric for measuring the overall performance of multi-object tracking algorithms. It comprehensively considers three types of errors: false detections, missed detections, and identity switches. The calculation formula of MOTA is as follows:

(21) $$MOTA=1-\frac{\sum_{t}\left(FN_{t}+FP_{t}+IDSW_{t}\right)}{\sum_{t}GT_{t}}$$

Among them, $FN_{t}$ is the number of missed detections in the t-th frame, $FP_{t}$ is the number of false detections, $IDSW_{t}$ is the number of identity switches, and $GT_{t}$ is the actual number of targets.

IDF1 is an important indicator for evaluating the consistency of identity preservation in multi-object tracking. The calculation formula of IDF1 is as follows:

(22) $$IDF1=\frac{2\times IDTP}{2\times IDTP+IDFP+IDFN}$$

Among them, IDTP (true positives) represents the number of correctly matched identities, IDFP (false positives) represents the number of incorrectly matched identities, and IDFN (false negatives) represents the number of missed identities.

In order to reflect the multi-object tracking performance of the optimized model more intuitively, a comparison is made among the traditional target tracking algorithms SORT and DeepSORT and the improved CNN-GRU. To maintain the uniqueness of variables in the comparative experiments, the detector used in the experiment process is DETR, and the performance comparison on the test set is shown in Table 2.

As can be seen from Table 2, the accuracy of the hybrid neural network has increased by 21.7% and 6.2%, respectively, compared with the SORT and DeepSORT trackers, which indicates that CNN-GRU can handle the nonlinear motion states of remote sensing targets better and has a significant improvement in multi-object tracking performance compared with traditional target tracking algorithms. Moreover, this paper also compares the impact of remote sensing images before and after super-resolution reconstruction on multi-object tracking (CNN-GRU). The results show that the accuracy and IDF1 value of the images with super-resolution reconstruction have increased by 4.3% and 0.031, respectively, compared with those without super-resolution reconstruction. This demonstrates that the remote sensing images after super-resolution reconstruction can not only improve the resolution and display the detailed features of the targets in the images better but also effectively reduce the noise in the original images and improve the performance of the tracker. The super-resolution reconstruction network combined with the CNN-GRU network has increased the accuracy (MOTA) by 26% and 10.5%, respectively, compared to SORT and DeepSORT.

## 5. Conclusions

In view of the characteristics such as scene complexity and noise in remote sensing images, this paper redesigns the residual blocks, upsampling blocks, and loss functions of the super-resolution reconstruction network based on EDSR. As a result, compared with the traditional EDSR, the super-resolution reconstruction model has improved its feature extraction ability and further enhanced the details and texture of the reconstructed remote sensing images. Among them, the PSNR and SSIM indicators have been increased by 1.17 dB and 0.12, respectively. Traditional target tracking algorithms consider the shape similarity of targets too much while ignoring the nonlinear characteristics in the motion process of remote sensing targets. The hybrid neural network CNN-GRU proposed in this paper regards the motion of remote sensing targets between frames as a time series prediction problem, thus effectively capturing the nonlinear characteristics of the inter-frame motion. It can effectively handle situations such as large variations in the spatial scale of targets and complex environments in remote sensing images. The experimental results show that the accuracy of the hybrid neural network proposed in this paper has increased by 21.7% and 6.2%, respectively, compared with the traditional SORT and DeepSORT trackers. Among them, the tracker with the super-resolution reconstruction module has increased its accuracy and IDF1 value by 4.3% and 0.031, respectively, compared with when it is not used, indicating that the super-resolution reconstruction network specially designed for remote sensing image processing proposed in this paper has greatly improved the accuracy of the target tracking algorithm. The shortcoming of the model lies in the fact that it does not extract features of the relative positions of remote sensing targets. It is necessary to improve the model’s ability to perceive the transformation of target positions in the next research work.

## Figures and Tables

**Table 1:** Comparison of ablation results.

Models	Residual Block	Upsample Block	Loss Function	PSNR	SSIM
EDSR	─	─	─	27.71	0.742
Improvement 1	√	─	─	28.09	0.828
Improvement 2	√	√	─	28.32	0.833
Improvement 3	√	√	√	28.88	0.862

**Table 2:** Track model performance comparisons.

Model	MOTA	IDF1
SORT	41.8%	0.389
DeepSORT	57.3%	0.564
CNN-GRU	63.5%	0.625
Super-resolution Reconstruction Network + CNN-GRU	67.8%	0.656

## Data Availability

The data sets used in this paper are all based on open source data sets.
